# Unsaturated Polyester Resin Nanocomposites Based on Post-Consumer Polyethylene Terephthalate

**DOI:** 10.3390/polym14081602

**Published:** 2022-04-14

**Authors:** Kirill Kirshanov, Roman Toms, Pavel Melnikov, Alexander Gervald

**Affiliations:** M.V. Lomonosov Institute of Fine Chemical Technologies, MIREA—Russian Technological University, 119571 Moscow, Russia; kirill_kirshanov@mail.ru (K.K.); toms.roman@gmail.com (R.T.); gervald@bk.ru (A.G.)

**Keywords:** PET, polyethylene terephthalate, oligoesters, unsaturated polyester resins, chemical recycling, glycolysis, interchain exchange, polymer-based nanocomposites, polymer matrix enhancement

## Abstract

A method for producing nanocomposites of unsaturated polyester resins (UPR) based on recycled polyethylene terephthalate (PET) as a matrix has been proposed. The upcycling method involves three successive stages: (1) oligoesters synthesis, (2) simultaneous glycolysis and interchain exchange of oligoesters with PET, (3) interaction of the obtained resins with glycol and maleic anhydride. UPRs were characterized by FTIR spectroscopy and gel permeation chromatography. The mechanical properties of nanocomposites obtained on the basis of these resins and titanium dioxide have been investigated. It has been shown that 1,2-propylene glycol units, despite their lower reactivity, significantly improve the properties of UPR. The most promising nanocomposite sample exhibited tensile strength 112.62 MPa, elongation at break 157.94%, and Young’s modulus 29.95 MPa. These results indicate that the proposed method made it possible to obtain nanocomposites with high mechanical properties based on recycled PET thus allowing one to create a valuable product from waste.

## 1. Introduction

Polymer nanocomposites are an intensively developing class of materials that consist of polymer matrices and nanomaterials distributed in them. Both thermoplastic and thermosetting polymers could be used as matrices. The nanomaterials used vary widely in geometry, with not only three-dimensional powders, but also linear fibers, nanotubes, and layered materials such as clay being used [1,2]. Modern nanocomposites have a wide variety of applications, including additive technologies (3D printing) [3].

Currently, environmental impact of nanocomposites application and production [4] and issues of sustainable polymer nanocomposites development [5] are of particular relevance. It is known that only condensation polymers could be fully recycled [6], with polyethylene terephthalate (PET) being able to be recycled even when contaminated [7]. Nanocomposites can be made based on thermoplastic polyesters with a high degree of polycondensation, such as PET. The presence of the nanofiller in the polyethylene terephthalate matrix was found to increase the glass transition temperatures and thermal stability of the material [8].

Although thermoplastic polyethylene terephthalate can be used for nanocomposites production directly, the utilization of thermoset post-consumer PET-based polyesters with lower viscosity can prove to be easier and more environmentally friendly [7]. For example, a good alternative to the use of pure recycled polyethylene terephthalate are unsaturated polyester resins (UPRs) [9]. These are double bond containing polyesters with a low degree of polycondensation. The main monomers used for UPRs synthesis are phthalic or maleic anhydride, phthalic, maleic or fumaric acid, ethylene glycol, diethylene glycol and 1,2-propylene glycol, with maleate and fumarate units containing double bonds. UPR composites contain monomers with double bonds (styrene or acrylates) and accelerator, and may contain also thickeners, fillers, reinforcing materials, mold release agents, low profile additives [9], and other additives, such as plasticizers. To cure the resins, a radical polymerization initiator is added.

Flame retardants and nanofillers can be distinguished among the nanomaterials introduced into unsaturated polyester nanocomposites, with nanofillers being the largest group [10,11,12]. The properties of the composites depend on the filler shape, size, aggregate size, surface characteristics, and degree of dispersion. One widely used inexpensive filler is calcium carbonate [13,14,15]. More expensive functional fillers are metal oxides [16], with titanium dioxide being one of the most usable. TiO_2_ nanoparticles have a number of specific properties: antimicrobial, photocatalytic, heavy metal ions sorption ability [17,18]. Filling of UPRs with nanoparticles proved to increase their thermal, mechanical, and anti-UV aging properties [19]. Known photocatalytic activity of titanium dioxide nanoparticles was also confirmed for UPR-based composites [20]. The effect of ether bonds count on the properties of the nanocomposite was studied using polyethylene glycols as an example [21]. An increase in the ether bonds content is shown to lead to the amplification of elastic properties and an increase in elongation at break. Other metal oxides used are zinc oxide [22,23,24,25] and iron oxides [16,26,27], mainly due to lower cost [28]. Silica [29,30,31] and silica-titania mixtures [32] are also widely applicable. The use of such widespread nanomaterials as clays [33,34,35], nanocellulose [36], carbon nanotubes [37,38,39], and nanodiamonds [40] has being investigated. It is also promising to use waste rubber [41], which is characterized by low cost [7] and special properties due to the content of carbon black [42].

**Figure 1:**
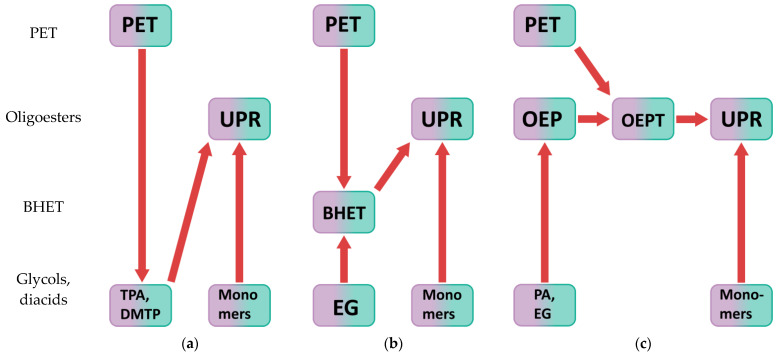
Different paths for the unsaturated polyester resins synthesis based on the products of post-consumer poly(ethylene terephthalate) chemical recycling: (**a**) PET hydrolysis or alcoholysis, where TPA—terephthalic acid, DMTP—dimethyl terephthalate; (**b**) PET heterogeneous glycolysis; (**c**) PET homogeneous glycolysis and interchain exchange.

UPS can be made from PET by chemical recycling [43,44]. There are many ways to chemically recycle polyethylene terephthalate [45,46,47]. Depending on the change in molecular weight, chemical recycling can be divided into directed degradation, interchain exchange, and solid-state polycondensation. Depending on the agent, hydrolysis, alcoholysis, acidolysis, glycolysis, and esterolysis are distinguished. In addition, chemical recycling can be carried out by various methods: heterogeneous recycling and homogeneous processing in solution or melt [7]. Figure 1 shows paths for obtaining UPRs from post-consumer polyethylene terephthalate chemical recycling products.

The first approach (Figure 1a) involves hydrolysis or alcoholysis of PET with terephthalic acid or dialkyl terephthalate production, respectively, followed by the synthesis of UPRs [45,46,47]. The second method comprises the synthesis of a resin based on bis(2-hydroxyethyl) terephthalate (BHET), which is a glycolysis product of polyethylene terephthalate, as shown in Figure 1b [45,46,47,48,49,50]. The most promising method is the chemical recycling of polyethylene terephthalate under the action of oligoesters with hydroxyl end groups [7,51,52]. It consists of simultaneous glycolysis and interchain exchange of PET and synthesized oligoester, followed by glycolysis and/or polycondensation with other co-monomers, as shown in Figure 1c. Preliminary polyethylene terephthalate molecular weight reduction under the action of oligoesters with hydroxyl end groups increases the efficiency of chemical recycling of polyethylene terephthalate, since such oligoesters:

- Contain sufficient concentration of hydroxyl groups to prevent the formation of cyclic oligomers and react readily;
- Are compatible with PET reaction in melt, implying co-solvent effect [7,53];
- Do not actively release low molecular weight compounds at temperatures above PET melting point even without an autoclave.

The process under consideration can be classified as upcycling, since it allows one to obtain value added products when blended with other materials [6].

Thus, the aim of this work is to investigate the process of unsaturated polyester resins of various composition using the chemical recycling of polyethylene terephthalate under the action of various oligoesters with hydroxyl groups and glycols, as well as to study the properties of TiO_2_/UPR nanocomposites based on the resins obtained.

## 2. Materials and Methods

### 2.1. Materials

Post-consumer transparent PET flakes with at least 95% of the main fraction, particle size from 5 to 10 mm were purchased from Tver Polymers Recycling Plant (Tver, Russia). Phthalic (PA) and maleic (MA) anhydrides, ethylene glycol (EG), diethylene glycol (DEG), and 1,2-propylene glycol (PG) were used as monomers; N,N-dimethylaniline and benzoyl peroxide were used as accelerator and initiator, respectively; titanium dioxide nanoparticles being colorless crystals of anatase structure with an average size of 25 nm were used as nanofiller. These reagents were purchased from Sigma Aldrich (St. Louis, MO, USA) and were used without further purification. Paraplex G-50 (Hallstar Industrial, Chicago, IL, USA) was used as plasticizer.

### 2.2. Unsaturated Polyester Resins Synthesis

#### 2.2.1. Saturated Oligoesters

Saturated oligoesters used as agents for PET simultaneous glycolysis and interchain exchange were synthesized as follows. Glycol was introduced into the molten phthalic anhydride at 140 °C in the ratio given in Table 1. The system was kept in an inert gas (nitrogen) flow at a constant temperature for 1.5 h with stirring at 100 rpm, followed by an increase in temperature to 190 °C and stirring up to 350 rpm for 3 h. After that, the reaction was carried out under a vacuum of 40 mbar for 3 h until the isolation of low molecular weight compounds was completed. As a result, samples OEP-1, ODEP-1 and OPP-1 were obtained from EG, DEG and PG, respectively.

#### 2.2.2. Simultaneous Glycolysis and Interchain Exchange of Oligoesters with PET

The process of simultaneous glycolysis and interchain exchange of PET and oligoesters was carried out at an equimolar ratio of PET and oligoesters units. Co-melting of PET and oligoesters at a temperature of 275 °C in an atmosphere of inert gas (nitrogen) was followed by lowering the temperature to 250 °C and conducting the reaction for 1.5 h with stirring at 50 rpm. As a result, samples OEPT-1, ODEEPT-1, and OPEPT-1 were obtained from OEP-1, ODEP-1, and OPP-1, respectively.

#### 2.2.3. Unsaturated Polyester Resins Synthesis

The synthesis of unsaturated polyester resins was carried out at a temperature of 150 °C under a vacuum of 40 mbar with stirring at 100 rpm for 1.5 h. The molar ratios of monomers and oligoesters units are given in Table 2.

### 2.3. Preparation of Nanocomposites

#### 2.3.1. Mixing UPR with Other Components

All composites were prepared from a mixture of 25 wt% styrene with 75 wt% of UPR samples. First, unsaturated polyester resin was dissolved in styrene, followed by addition of nanoparticles (5 wt%) and N,N-dimethylaniline accelerator taken in equimolar ratio to the initiator added in the next step. The mixture was kept on the Ultrasonic Processor (Cole-Parmer Instruments, Vernon Hills, IL, USA) for 2.5 h.

#### 2.3.2. Composite Curing

Benzoyl peroxide initiator was mixed with Paraplex G-50 plasticizer in a 2:1 weight ratio, followed by addition to UPR composite at a rate of 2 wt% of initiator relative to the resin. The mixture was stirred for 30 s to distribute the components by volume, and then it was immediately injected into molds. Curing took place within 24 h.

### 2.4. Characterization of PET and Oligoesters

#### 2.4.1. Fourier Transform Infrared Spectroscopy (FTIR)

The chemical compositions of PET and the resulting oligoesters were confirmed by the position of the characteristic bands in the FTIR spectra. The spectra were obtained by means of Spectrum 65 FT-IR spectrometer (Perkin Elmer, Waltham, MA, USA).

#### 2.4.2. Gel Permeation Chromatography (GPC)

Gel permeation chromatography (Gilson Inc., Middleton, WI, USA) with Agilent MIXED-E column, tetrahydrofuran as the mobile phase, and refractive index detector was used to determine the molecular weights of the component present in the analyzed samples. Measurements were made at the temperature of 25 °C and the flow rate of 1.0 mL/min. Narrowly dispersed polystyrene standards with Mp (peak molecular weight) 580; 1280; 2940; 10,110; 28,770 g/mol and polydispersity index no more than 1.12 were used for calibration (Agilent, Santa Clara, CA, USA).

#### 2.4.3. Oligoesters Synthesis Conversion

Oligoesters Synthesis Conversion is determined using the Carothers equation [54]. The degree of polycondensation was determined by Equation (1):

(1) $$P=\frac{M_{n}}{{\mathrm{MM}}_{\mathrm{av}}}\;,$$

where M_n_ is the number average molecular weight, g/mol, determined by GPC, MM_av_ is the unit average molecular weight, g/mol, taking into account the mole fraction.

The unbalance factor was determined by Equation (2):

(2) $$q=\frac{N_{X0}}{N_{Y0}}\;,$$

where N_X0_ and N_Y0_ are the amounts of glycols and diacids in the initial monomer mixture, with N_X0_ ≥ N_Y0_.

The oligoester synthesis conversion was calculated by adjusting the parameter in Equation (3):

(3) $$P=\frac{1+q}{1+q\times \left(1-2\propto \right)}\;,$$

where P is the degree of polycondensation determined by Equation (1), q is the unbalance factor calculated using Equation (2), and α is the desired conversion, %.

#### 2.4.4. Color

The color of the samples was determined using Gardner scale in accordance with ASTM D1544 using BYK Gardner Liquid Color Standarts (BYK Additives & Instruments, Wesel, Germany).

### 2.5. Characterization of UPR Cures

#### 2.5.1. Density Measurement

Density of cured composites was determined using immersion method according to ISO 1183-1:2019 using distilled water at 25 °C.

#### 2.5.2. Test Method for Tensile Properties

Mechanical properties were determined according to ASTM D638. The cross-sectional dimensions of the specimens were 10 × 5 mm, the stretching rate was 5 mm/min. Instron 5942 testing machine (Instron, Norwood, MA, USA) and Instron Bluehill software were used for tensile strength (MPa), elongation at break (%), and Young’s modulus (MPa) measurements. According to the test method, a standard error of no more than 10% of the measured parameter is achieved with an elongation of more than 20%.

## 3. Results and Discussion

### 3.1. PET-Flakes Characterization

**Figure 2:**
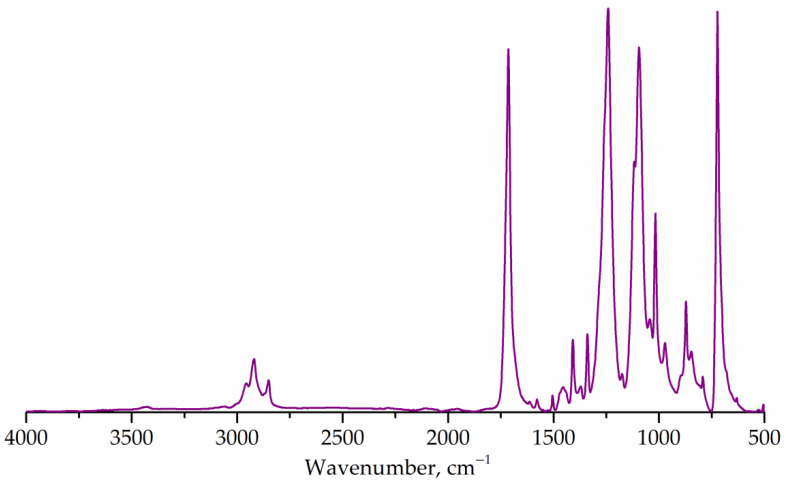
FTIR spectrum of PET flakes.

Figure 2 shows the FTIR spectrum of the untreated PET flakes. The main absorption bands corresponding to the PET structure were determined at 1715 cm^–1^ (carbonyl, stretching), 1243 cm^–1^ (ester group, stretching), 1176 and 1116 cm^–1^ (1,4-substituted ring) [7,55]. The correlation coefficient with the known spectrum of polyethylene terephthalate was 99.3%, which confirms that the sample represents a partially crystalline PET [55].

### 3.2. Oligoesters Characterization

#### 3.2.1. OEP-1, ODEP-1, and OPP-1 Properties

**Figure 3:**
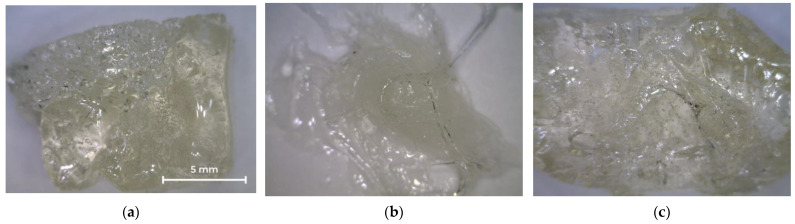
Photos of OEP-1, ODEP-1, and OPP-1 samples: (**a**) OEP-1; (**b**) ODEP-1; (**c**) OPP-1.

Figure 3 shows photographs of the oligoester agents obtained by glycolysis and interchain exchange. All saturated resins are transparent, so their color can be assessed on the Gardner scale (Table 3). OEP-1 and OPP-1 samples are brittle solids at room temperature (Figure 3a,c), while ODEP-2 is a viscous, sticky resin (Figure 3b).

**Figure 4:**
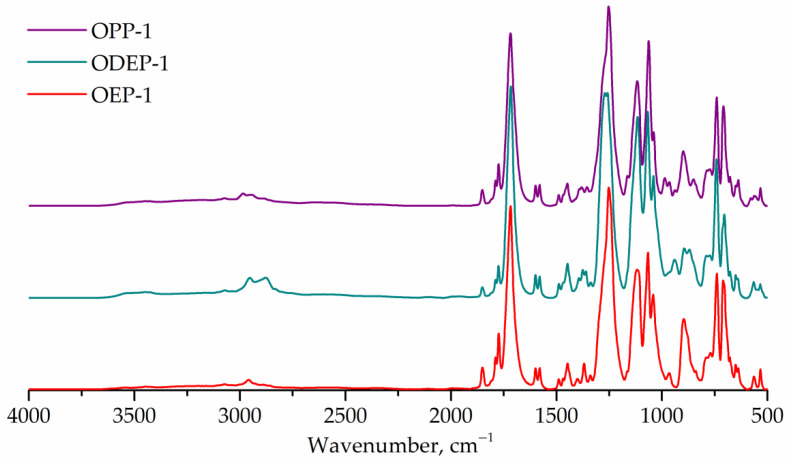
FTIR spectra of OEP-1, ODEP-1, and OPP-1 samples.

The FTIR spectra of the obtained saturated polyester resins are shown in Figure 4. All samples exhibit bands at 1715 cm^–1^ (carbonyl, stretching) and 1243 cm^–1^ (ester group, stretching). The FTIR spectrum of the ODEP-1 sample contains bands corresponding to the ether bond of diethylene glycol: 1270 cm^–1^ (C-O-C, assym. stretching), 939 cm^–1^ (C-O-C, sym. stretching). The bands characterizing the CH_3_-group overlap with other ones, so they cannot be marked. The ratios of the bands corresponding to the hydroxyl (3350 cm^–1^) and ester (1715 cm^–1^) groups are the same for all three samples.

**Figure 5:**
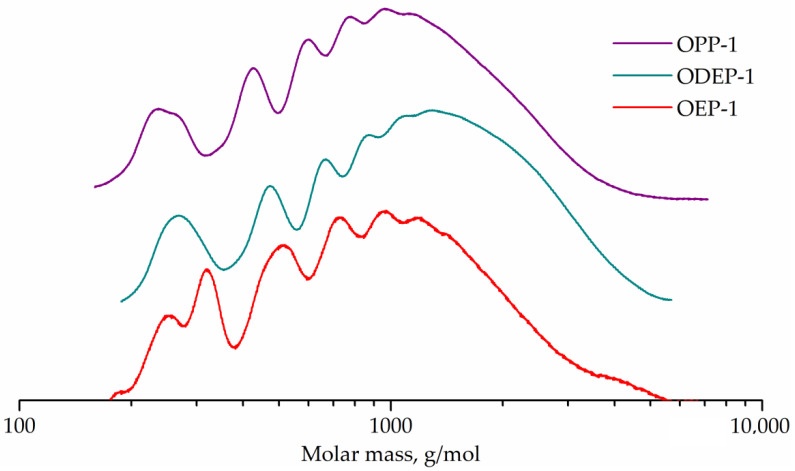
GPC curves of OEP-1, ODEP-1, and OPP-1 samples.

GPC curves for OEP-1, ODEP-1, and OPP-1 samples are shown in Figure 5.

The GPC curve of sample OEP-1 has a peak (257 g/mol) with a shoulder (195 g/mol) corresponding to phthalic acid ethylene glycol diester and monoester. The adjacent strong peak (337 g/mol) seems to correspond to phthalic acid diethylene glycol diester, which is known to form in polycondensation processes with ethylene glycol [7]. Other peaks (504, 727, 945, and 1198 g/mol) correspond to oligomers.

The GPC curve for the diethylene glycol-containing ODEP-1 sample has a peak (268 g/mol) with a shoulder (315 g/mol) corresponding to phthalic acid diethylene glycol monoester and diester, and peaks of oligomers (474, 667, 882, and 1089 g/mol).

The OPP-1 sample curve exhibits the peak of phthalic acid 1,2-propylene glycol monoester (236 g/mol) with a shoulder of diester (263 g/mol) as well as peaks of oligomers (426, 594, 765, and 933 g/mol).

Peak molecular weights for OEP-1, ODEP-1, and OPP-1 samples differ from the calculated for corresponding monomers and oligomers ones, since polystyrene standards were used. The GPC curves were used to determine the molecular weight characteristics, followed by the conversion calculation (Table 3).

The GPC curves and calculated conversions of more than 95% for all resins confirm the formation of oligomers, which is expected in the polycondensation of a mixture of monomers with an unbalance factor of 0.8. The color of the samples is in line with theoretical expectations, since the end groups of ethylene glycol (in sample OEP-1) easily lead to the formation of known chromophores, both polyenaldehydes and polyenes [56].

#### 3.2.2. OEPT-1, ODEEPT-1, and OPEPT-1 Properties

**Figure 6:**
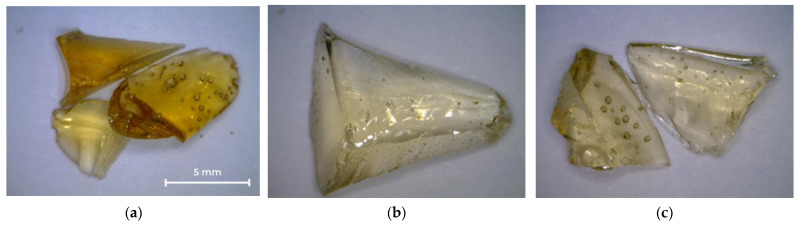
Photos of OEPT-1, ODEEPT-1, and OPEPT-1 samples: (**a**) OEPT-1; (**b**) ODEEPT-1; (**c**) OPEPT-1.

Photos of PET-based saturated polyester resins obtained by the reaction of PET and OEP-1, ODEP-1, and OPP-1 oligoesters are shown in Figure 6. The resulting samples are transparent solids.

**Figure 7:**
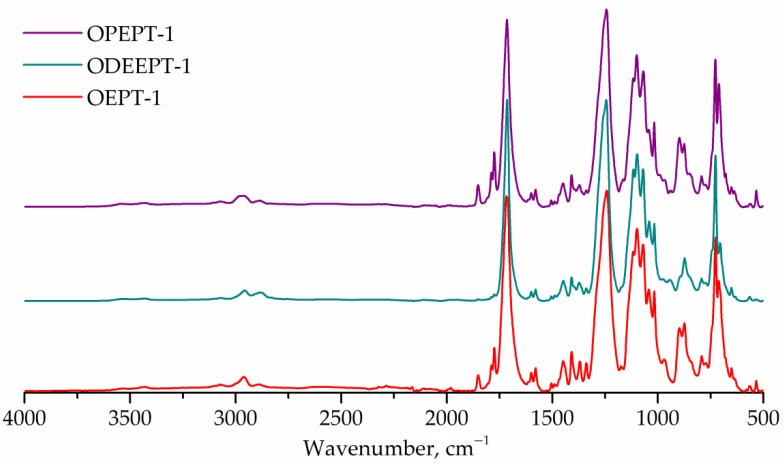
FTIR spectra of OEPT-1, ODEEPT-1, and OPEPT-1 samples.

FTIR spectra of terephthalate-containing resins are shown in Figure 7. FTIR spectra for PET-based saturated polyester resins exhibit peaks at 1176 and 1116 cm^–1^ (1,4-substituted ring), same as in polyethylene terephthalate spectra [7,55].

**Figure 8:**
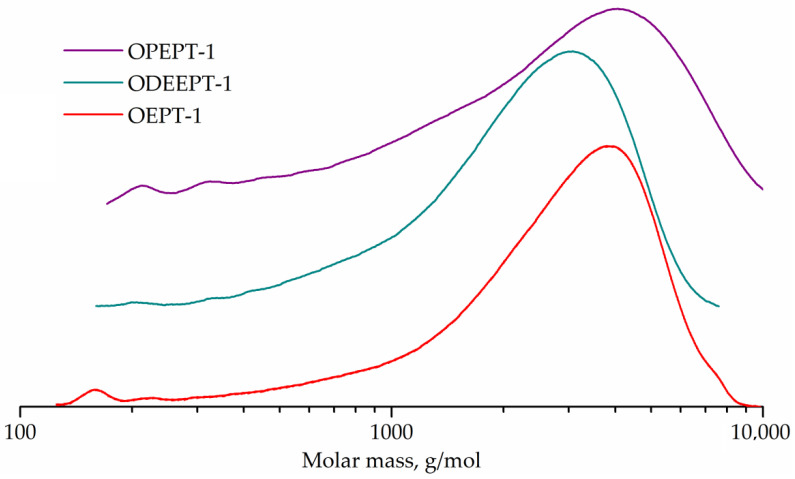
GPC curves of OEPT-1, ODEEPT-1, and OPEPT-1 samples.

Figure 8 represents the GPC curves. The GPC curves of all three samples are flat peaks corresponding to higher oligomers, with only weak peaks of monomers, dimers and trimers being distinguishable. The peak molecular weight was 3769 g/mol for OEPT-1 sample, 3184 g/mol for ODEEPT-1, and 4031 g/mol for OPEPT-1.

The end groups of polyethylene terephthalate were not taken into account during the conversion calculation, since even post-consumer polyethylene terephthalate has a much higher molecular weight than oligoesters (number average molecular weight of about 26,000 g/mol [57]). Characteristics and conversion of samples are shown in Table 4.

In contrast to the polycondensation of OEP-1, ODEP-1 and OPP-1 oligoesters, where the reaction with unbound 1,2-propylene glycol reaches a noticeably lower conversion, the conversion in this process turned out to be practically independent of the chemical composition of the oligomer at an equal concentration of terminal hydroxyl groups (within 0.2% range). All samples acquired a more intense color, which is in line with expectations for the second heat treatment cycle. Both the absence of the dependence of the conversion on the composition and the more intense coloration are obviously related to the higher temperature of the described process. Differences in the colors of the samples correspond to the assumption made in Section 3.2.1.

#### 3.2.3. Unsaturated Polyester Resins Properties

**Figure 9:**
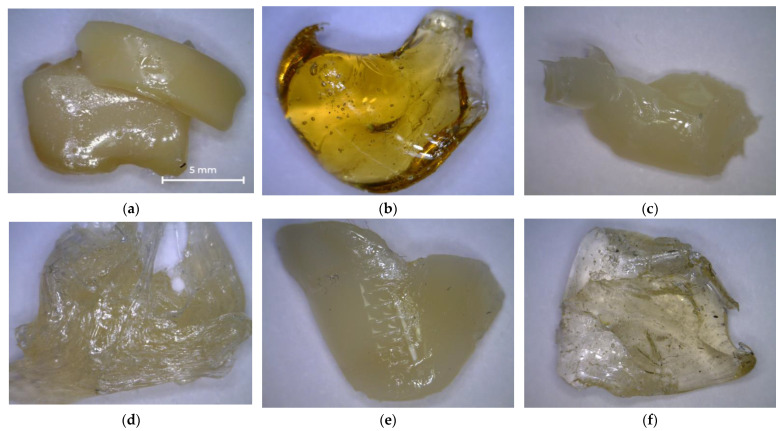
Photos of UPR samples: (**a**) UPR-1; (**b**) UPR-2; (**c**) UPR-3; (**d**) UPR-4; (**e**) UPR-5; (**f**) UPR-6.

Photos of the resulting unsaturated polyester resins are shown in Figure 9. Samples treated with diethylene glycol appeared to be opaque sticky and highly viscous resins (Figure 9a,c,e), while the remaining samples persisted to be transparent (Figure 9b,d,f).

**Figure 10:**
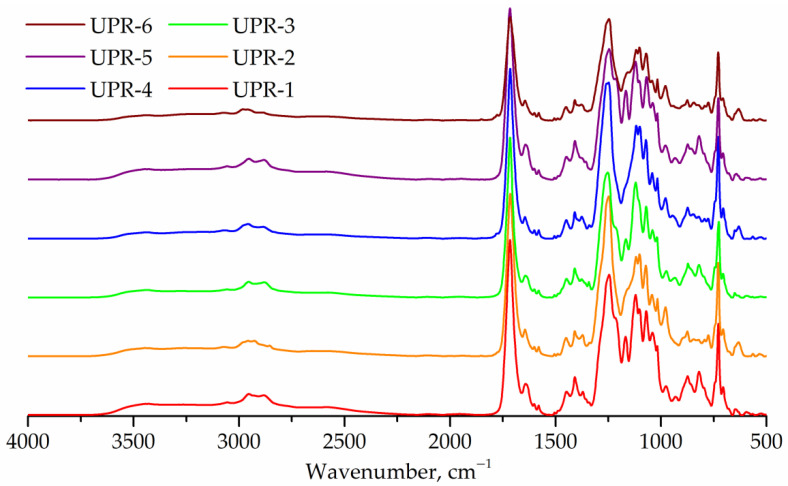
FTIR spectra of UPR-1, UPR-2, UPR-3, UPR-4, UPR-5, and UPR-6 samples.

IR spectra of UPRs are shown in Figure 10. The spectra of all samples show an elevated region of 2300–3700 cm^−1^, which corresponds to the hydroxyl end groups. The IR spectra of UPR-2, UPR-4, and UPR-6 resins show a band at 1780 cm^−1^ (anhydride, stretching) corresponding to maleic anhydride.

**Figure 11:**
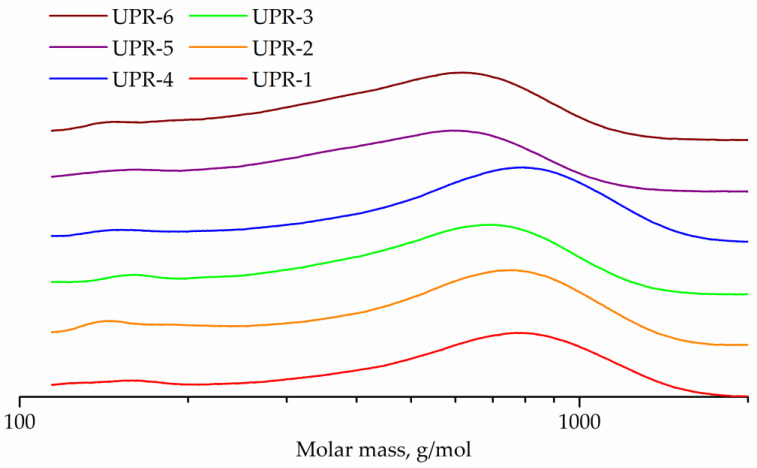
GPC curves of UPR-1, UPR-2, UPR-3, UPR-4, UPR-5, and UPR-6 samples.

The GPC curves of the obtained UPRs are shown in Figure 11. In addition to the main peaks of oligomers, all curves contain weak peaks in the range 147–167 g/mol, corresponding to maleic acid ethylene, diethylene or 1,2-propylene glycol monoester, respectively. Peak molecular weights for UPR-1, UPR-2, UPR-3, UPR-4, UPR-5, and UPR-6 samples are 780, 753, 694, 794, 590, and 620, respectively.

The conversion cannot be calculated, because the reactions of glycolysis of OEPT-1, ODEEPT-1, and OPEPT-1 oligoesters and polycondensation of diethylene or 1,2-propylene glycols and maleic anhydride occur simultaneously in this process. Molecular weight characteristics of samples of UPRs are shown in Table 5.

Unbound 1,2-propylene glycol is less active in the reaction than diethylene glycol, as evidenced by the transparency of samples UPR-2, UPR-4, and UPR-6, and by the presence of peaks corresponding to maleic anhydride in the FTIR spectra of these samples. Lower molecular weights of samples UPR-4 and UPR-6 compared to UPR-3 and UPR-5 were also observed. The same dependencies were observed for the polycondensation reaction (Section 3.2.1).

### 3.3. Nanocomposites Properties

The first step in the preparation of unsaturated polyester resin nanocomposite is the dissolution of UPR in styrene. Samples UPR-1, UPR-3, and UPR-5 dissolved readily, while sample UPR-2 was completely insoluble. Samples UPR-4 and UPR-6 formed physical gels when dissolved in styrene, with UPR-4 remaining in gel form even when excess styrene was added. Since UPR-4 and UPR-5 have the same composition of monomer mixtures, it can be concluded that the molecular weight has a significant effect on the properties of the unsaturated polyester resin. It should be noted that the molecular weight of the product in the process under study depended on whether the monomers were used in unbound form or as an oligomer at different stages.

**Figure 12:**
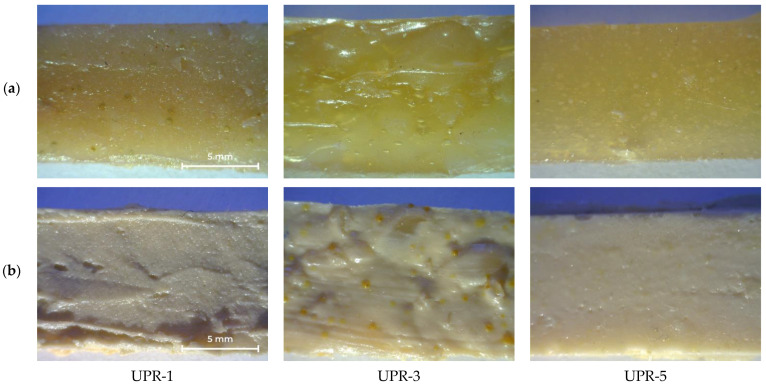
Photos of cured UPR-1, UPR-3, and UPR-5 samples: (**a**) without TiO_2_; (**b**) with TiO_2_.

The appearance of cured UPRs with or without titanium dioxide is shown in Figure 12. The UPR-3 sample is highly heterogeneous, as evidenced by the yellow inclusions (Figure 12b). Density and mechanical properties are shown in Table 6, stress-strain curves of UPR samples are given in Supplementary Materials (Figure S1).

The density of all samples is approximately the same, however, it slightly increases with an increase in the content of diethylene glycol and 1,2-propylene glycol units. The addition of 5 wt% titanium dioxide also increases the density of the nanocomposite.

Tensile strength for samples without titanium dioxide increases in the series UPR-1 < UPR-3 < UPR-5. Nanocomposites based on UPR-1 and UPR-5 exhibit increase in tensile strength (by 2.2 and 117.2 %, respectively), however UPR-3 based sample does not follow the same pattern (71.3% decrease). This may be due to its heterogeneity, the influence of titanium dioxide [16], or the content of ether bonds [21]. The Young’s modulus follows the same pattern (increased by 33.9 and 210.4 % for UPR-1 and UPR-5, respectively). UPR-5 samples, especially TiO_2_-containing one, have outstanding strength properties, surpassing the studied analogues [16,21].

The elongation at break increases with the addition of titanium dioxide (by 21.2, 55.3, and 24.3 % for UPR-1, UPR-3, and UPR-5, respectively). Achieved values are approximately the same for samples UPR-1 and UPR-3, with sample UPR-3 without TiO_2_ having slightly less elongation than UPR-1. This can be caused both by the inhomogeneity of the samples and by the influence of ether bonds, which are known to reduce elongation at break above a certain concentration [21]. The elongation at break for the UPR-5 sample increases dramatically compared to the other ones. It is almost 5 times greater than that of the UPR-1 and UPR-3 composites, and more than 7 times as compared to UPR-3 without the addition of titanium dioxide.

## 4. Conclusions

A method for the preparation of unsaturated polyester resins based on recycled polyethylene terephthalate has been studied. It includes successive stages of oligoesters synthesis, their simultaneous glycolysis and interchain exchange with PET, simultaneous glycolysis of the resulting PET-based oligoesters, and polycondensation with maleic anhydride. In the first and third processes, which were carried out in the temperature range of 140–190 °C, the reactivity of 1,2-propylene glycol turned out to be the lowest, yielding the 95.96% conversion during the synthesis of the oligoester, higher molecular weights, and resulting in the presence of bands corresponding to maleic anhydride in the spectrum after glycolysis. However, in the case of combined glycolysis and interchain exchange, which occur in the temperature range of 250–275 °C, the conversion turned out to be practically uninfluenced by oligoester composition. The Gardner color value for the samples containing the highest proportion of 1,2-propylene glycol was the lowest.

TiO_2_-based nanocomposites utilizing resin samples that do not contain additional units of ethylene glycol to those present in the original polyethylene terephthalate surpassed the samples produced from ethylene glycol-containing oligoether. The Tensile strength and Young’s modulus differ by more than an order of magnitude. The elongation at break increased significantly for samples containing 1,2-propylene glycol. The addition of titanium dioxide nanoparticles in most cases increases the mechanical properties and density, with the most significant increase occurring for the 1,2-propylene glycol-containing sample.

Thus, the most rational way to obtain resins utilizing post-consumer polyethylene terephthalate as a matrix for nanocomposites is based on combined glycolysis and interchain exchange of a 1,2-propylene glycol-containing oligomer with PET, followed by glycolysis with diethylene glycol and polycondensation with maleic anhydride. We assume that this chemical recycling process has lower moisture requirements than thermomechanical recycling. The obvious disadvantages include a large number of stages. As in other polycondensation processes, the high purity of the feedstock is important for the synthesis of oligoesters at the first stage. There are also temperature limitations due to degradation processes. Presumably, the temperature should not exceed 280 °C. The ratios of PET, oligomers, glycols and anhydride at all stages, as well as the temperature conditions of the process, are worth further investigation in order to be implemented in the industry.

## Figures and Tables

**Table 1:** Molar ratios of OEP-1, ODEP-1, and OPP-1 samples synthesis reagents.

Reagents	OEP-1	ODEP-1	OPP-1
PA	4	4	4
EG	5	0	0
DEG	0	5	0
PG	0	0	5

**Table 2:** Molar ratios of UPR-(1–6) samples synthesis reagents.

Reagents	UPR-1	UPR-2	UPR-3	UPR-4	UPR-5	UPR-6
MA	1	1	1	1	1	1
OEPT-1	2	2	0	0	0	0
ODEEPT-1	0	0	2	2	0	0
OPEPT-1	0	0	0	0	2	2
DEG	1	0	1	0	1	0
PG	0	1	0	1	0	1

**Table 3:** Characteristics of OEP-1, ODEP-1, and OPP-1 samples: number average (Mn) and weight average (Mw) molecular weights, polydispersity index (PDI), conversion, and color.

Sample	Mn, g/mol	Mw, g/mol	PDI	Conversion, %	Color
OEP-1	684	1100	1.61	97.65	3
ODEP-1	835	1368	1.64	97.04	1
OPP-1	667	1096	1.64	95.96	1

**Table 4:** Characteristics of OEPT-1, ODEEPT-1, and OPEPT-1 samples: number average (Mn) and weight average (Mw) molecular weights, polydispersity index (PDI), conversion, and color.

Sample	Mn, g/mol	Mw, g/mol	PDI	Conversion, %	Color
OEPT-1	1519	3053	2.01	96.81	9
ODEEPT-1	1786	2594	1.45	97.01	5
OPEPT-1	1600	3272	2.04	96.86	4

**Table 5:** Characteristics of UPR-1, UPR-2, UPR-3, UPR-4, UPR-5, and UPR-6 samples: number average (Mn) and weight average (Mw) molecular weights, polydispersity index (PDI) and color.

Sample	Mn, g/mol	Mw, g/mol	PDI	Color
UPR-1	446	657	1.47	Cloudy
UPR-2	416	640	1.54	10
UPR-3	410	574	1.40	Cloudy
UPR-4	492	692	1.41	7
UPR-5	354	490	1.38	Cloudy
UPR-6	381	517	1.36	4

**Table 6:** Density and mechanical properties of UPR samples.

Resin Sample	Nanofiller Content, wt%	Density, g/mm^3^	Tensile Strength, MPa	Elongation at Break, %	Young’s Modulus, MPa
UPR-1	0	1.22	3.61	27.14	0.62
	5	1.26	3.69	32.89	0.83
UPR-2	0	Insoluble in styrene			
	5				
UPR-3	0	1.24	34.32	21.68	7.33
	5	1.28	9.85	33.66	1.99
UPR-4	0	Forms a physical gel			
	5				
UPR-5	0	1.26	51.86	127.06	9.65
	5	1.29	112.62	157.94	29.95
UPR-6	0	Forms a physical gel			
	5				

## Data Availability

Not applicable.

## Supplementary Materials

The following supporting information can be downloaded at: https://www.mdpi.com/article/10.3390/polym14081602/s1, Figure S1: Stress-strain curves of UPR samples.
