# Supplementary material for: Unsaturated Polyester Resin Nanocomposites Based on Post-Consumer Polyethylene Terephthalate
Source: Polymers (Basel). 2022 Apr 14;14(8):1602. doi: 10.3390/polym14081602 (PMC9025439; doi:10.3390/polym14081602)
Supplement: Supplementary file 1 [file polymers-14-01602-s001.zip › polymers-1666349-supplementary.pdf]

Supplementary materials

Unsaturated Polyester Resin Nanocomposites Based on Post-Consumer Polyethylene Terephthalate

Kirill Kirshanov, Roman Toms, Pavel Melnikov * and Alexander Gervald

M.V. Lomonosov Institute of Fine Chemical Technologies, MIREA—Russian Technological University, 119571 Moscow, Russia; kirill_kirshanov@mail.ru (K.K.); toms.roman@gmail.com (R.T.); gervald@bk.ru (A.G.)

* Correspondence: melnikovsoft@mail.ru

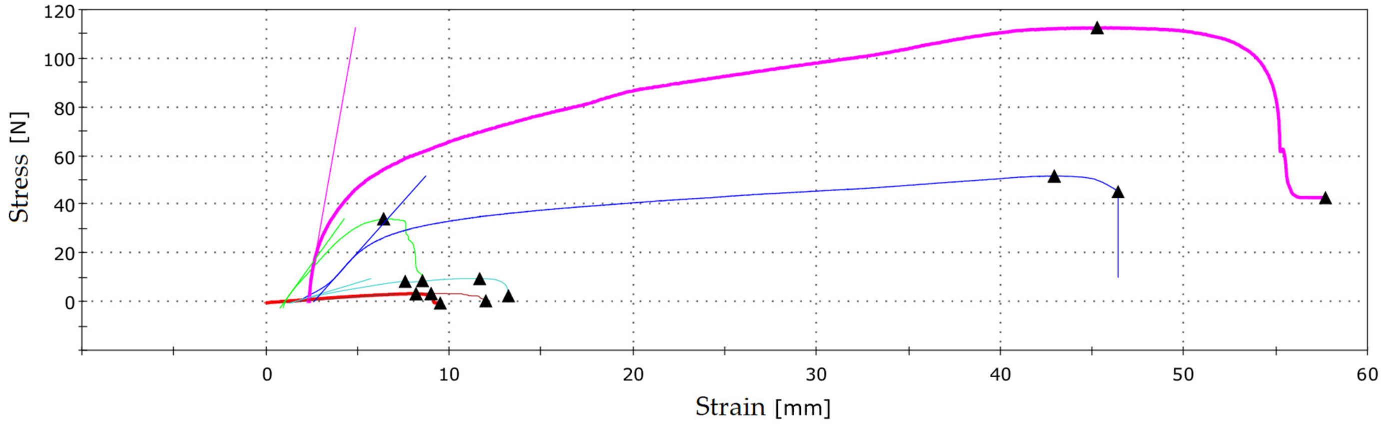

Figure S1. Stress-strain curves of UPR samples: (1) UPR-1 without TiO_2; (2) UPR-1 with TiO_2; (3) UPR-2 without TiO_2; (4) UPR-2 with TiO_2; (5) UPR-3 without TiO_2; (6) UPR-3 with TiO_2.
